# Use of antibodies against Epstein–Barr virus nuclear antigen 1 for detection of cellular proteins with monomethylated arginine residues that are potentially involved in viral transformation

**DOI:** 10.1007/s00705-024-06172-7

**Published:** 2024-11-08

**Authors:** Christian Graesser, Ruth Nord, Heinrich Flaswinkel, Elisabeth Kremmer, Eckart Meese, Karolina Magdalena Caban, Thomas Fröhlich, Friedrich A. Grässer, Martin Hart

**Affiliations:** 1 Institute of Virology, Saarland University Medical School, Kirrbergerstraße, Haus 47, D-66421 Homburg/Saar, Germany; 2 Department of Biology II, Ludwigs-Maximilians-Unversität (LMU) Munich, Butenandtstraße 1, 81377 Munich, Germany; 3 Institute of Human Genetics, Saarland University (USAAR), Kirrbergerstraße, Haus 60, 66421 Homburg/Saar, Germany; 4 Laboratory for Functional Genome Analysis (LAFUGA), Gene Center, LMU Munich, Feodor-Lynen-Straße 25, 81377 Munich, Germany; 5 Center of Human and Molecular Biology (ZHMB), Institute of Human Genetics, Saarland University (USAAR), Kirrbergerstraße, Haus 60, Building 60, D-66421 Homburg/Saar, Germany

## Abstract

Epstein–Barr virus nuclear antigen 1 (EBNA1) contains two arginine-glycine (RG) repeats that contain symmetric/asymmetric dimethylarginine (SDMA/ADMA) and monomethylarginine (MMA) residues. We generated mouse monoclonal antibodies directed against a monomethylated GRGRGG-containing repeat located between amino acids 328 and 377 of EBNA1. In addition to detecting MMA-modified EBNA1, we also had the goal of identifying cellular proteins that bind to MMA-modified EBNA1 in EBV-positive Raji cells. Furthermore, we hypothesized that antibodies against MMA-modified EBNA1 might also recognize cell factors that use an MMA-modified surface structure similar to that of EBNA1 to bind to their common targets. Using a combination of immunoprecipitation and mass spectrometry, we identified a number of such cellular proteins, including SNRPD1-3, ALY/REF, RPS15, DIDO1, LSM12, LSM14A, DAP3, and CPSF1. An NACA complex protein that was shown previously to bind to the glycine-alanine repeat of EBNA1 was also identified. The proteins identified in this study are involved in splicing, tumorigenesis, transcriptional activation, DNA stability, and RNA processing or export.

**Supplementary Information:**

The online version contains supplementary material available at 10.1007/s00705-024-06172-7.

## Introduction

The oncogenic Epstein–Barr virus (EBV) has been detected in nasopharyngeal carcinoma (NPC), nasal NK/T-cell lymphoma (NKTL), Burkitt’s lymphoma (BL), Hodgkin’s lymphoma (HL), gastric carcinoma (GC), and posttransplant lymphoproliferative disease (PTLD) samples from immunosuppressed patients [16] and appears to play a role in multiple sclerosis [20]. Epstein–Barr virus nuclear antigen 1 (EBNA1) is essential for cell transformation and maintenance of episomal EBV DNA in infected cells and is the only viral protein present in all types of infected cells [6].

EBNA1 contains a glycine-alanine (GA) repeat that varies in length among viral strains and is important for immune evasion during primary infection [5]. It also contains arginine-methylated arginine-glycine (RG) repeats that are involved in RNA and DNA binding [19] (Supplementary Fig. S1). The RG repeats are located between the N-terminal amino acids 34–52 (LR1) and the C-terminal amino acids 328–377 (LR2), which play a critical role in replication of the viral episome by targeting EBNA1 to the origin recognition complex (ORC) [14].

Posttranslational arginine methylation in proteins plays a role in RNA binding, protein‒protein interactions, transcriptional regulation, signal transduction, chromatin remodeling, and DNA repair. The arginine methyl transferases PRMT-1, -2, -3, -4 (CARM1), -6, and − 8 generate either monomethylarginine (MMA) or asymmetric dimethylarginine (ADMA) residues, PRMT5 and PRMT9 generate either MMA or symmetric dimethylarginine (SDMA) residues, and PRMT7 appears to preferentially generate MMA residues [26].

Previously, we showed that a monoclonal antibody (mAb) directed against the MMA-modified RG repeat of EBV-encoded nuclear antigen 2 (EBNA2) also reacted with the N-terminal RG repeat of EBNA1 (aa 34–52) [1]. While the EBNA2 RG repeat and the N-terminal RG repeat of EBNA1 mainly consist of RGRG sequences, the C-terminal RG repeat of EBNA1 (aa 328–377) also contains RGRGG sequences. We therefore generated monoclonal antibodies against two synthetic MMA-modified peptides derived from the C-terminal RG repeat of EBNA1 to identify cellular factors that bind to MMA-modified EBNA1. As methylated RG repeats serve as contact surfaces to bind to target proteins and thereby affect downstream pathways [26], an additional hypothesis of this study was that these antibodies would react with cellular proteins whose function is mimicked by EBNA1. The detection of such proteins might help to identify previously unknown pathways that are targeted during EBV-mediated transformation.

## Materials and methods

### Cell lines and transfection

HEK 293E1 cells, which express EBNA1 of EBV strain B95-8, were obtained from Aloys Schepers, Helmholtz Zentrum München, Munich, Germany, and were maintained in DMEM (Sigma‒Aldrich, #D0822, Merck, Darmstadt, Germany) supplemented with 0.1 mg of G418 (Sigma‒Aldrich #A1720, Merck, Darmstadt, Germany) per mL [14]. The EBV-positive Raji (ATCC: CCL-86), M-ABA, and P3HR-1 (ATCC: HTB-62) [1] cells were grown in RPMI-1640 medium (Sigma‒Aldrich #R8758, Merck, Darmstadt, Germany) supplemented with 10% fetal bovine serum (FBS; #S0615, Biochrom, Berlin, Germany) and antibiotics (40 IU of penicillin and 50 µg of streptomycin [Sigma‒Aldrich #P4333, Merck, Darmstadt, Germany], 1 IU of neomycin sulfate [#1405-10-3; Roth, Karlsruhe, Germany], and 90 IU of nystatin [#700114-0006; Fagrom, Barsbüttel, Germany]) per mL. The EBV-transformed marmoset cell line M-ABA was originally obtained from Beverly E. Griffin, Imperial College of Medicine, London, UK [7].

### Animals and antibodies

C57BL6J mice were maintained at the animal facility at the faculty of Biology, LMU Munich, in accordance with German Animal Welfare Legislation and that of the Government of Upper Bavaria, Germany (Gz: 55.2-1-54-2532.0-12-2016). For immunization, the peptides Cys-grgrggsggrgrggsggrgrggsggr and Cys-GGsggrgrggsggrrgrgrerARGGSRE containing monomethylarginine (R, MMA) residues corresponding to aa 328–377 (grgrggsggrgrggsggrgrggsggrrgrgrerARGGSRE) were coupled to ovalbumin via their Cys residues. Monoclonal antibodies were produced as described previously [9] and screened by ELISA for their ability to recognize the unmethylated (NMA) peptide or the methylated peptide. Clones 5C7, 7C10, and 1H7 (mouse IgG2c), which reacted exclusively with the MMA-containing peptides, were used for further analysis. Because these antibodies reacted with both of the methylated peptides described above, peptides corresponding to the overlapping GRGRGG sequence were used for release experiments (see below). All of the peptides used in this study were synthesized by Peps4LS GmbH (Heidelberg, Germany). The rat mAb 1H4, which recognizes EBNA1, was described previously [8]. Mouse mAb (IgG2c) against HSV-1 ICP8 (unpublished) was used as a control.

### Cell lysis and immunoprecipitation

Immunoprecipitation with the mAbs 5C7, 7C10, and 1H7 was performed as described previously [1]. In brief, protein A Sepharose beads (Protein A Sepharose 4 FastFlow #17-5280, GE Healthcare, Freiburg, Germany) were incubated overnight with 1.6 mL of mAb 5C7 or 1.6 ml of the isotype control antibody at 4°C and washed with lysis buffer (LB) (25 mM Tris-HCl [pH 7.4], 150 mM KCl, 2 mM EDTA, 0.5% IGEPAL-CA360 [Sigma‒Aldrich #18896, Merck, Darmstadt, Germany], and protease inhibitors [Roche Complete mini, #4693159001, Merck, Darmstadt, Germany]). A total of 1.5 × 10^7^ Raji cells were suspended in 1 ml of LB for 30 min, centrifuged at 20,000 × *g* for 30 min at 4°C, washed with a buffer containing 50 mM Tris-HCl (pH 7.4), 300 mM KCl, 1 mM MgCl_2_, and 0.5% IGEPAL, and analyzed by immunoblotting and enhanced chemiluminescence (ECL) (#6883, Cell Signaling Technology, Danvers, Massachusetts, USA) detection [1]. For release experiments with the unmethylated GRGRGG and MMA-modified GRGRGG peptide, mAb-5C7-conjugated beads loaded with Raji cell extracts were incubated with the peptides at 0.1 mg/mL in 10 µL of washing buffer (see above), 10% of the released fraction was subjected to Western blotting [1], and the remainder was used for mass spectrometry (see below).

### Mass spectrometry analysis

Samples from the protein release experiments were boiled with an equal amount of 2x Laemmli buffer and loaded onto a polyacrylamide gel (TGX Stain-Free Precast Gels 4–20%, Bio-Rad, Hercules, CA, USA, # 4568091), and proteins were separated by the application of 200 V for 3 min. The gels were stained with InstantBlue (#AB119211, Abcam, Cambridge, UK), and the protein-containing regions were excised. For reduction and alkylation, the gel pieces were incubated first in 45 mM dithiothreitol (30 min, 55°C) and then in 100 mM iodoacetamide (2 × 15 min, at room temperature). The gel slices were minced, and 70 ng of modified porcine trypsin (#V5111, Promega, Fitchburg, WI, USA) was added. After overnight digestion at 37°C, peptides were extracted with 70% acetonitrile and dried in a SpeedVac vacuum concentrator. The samples were dissolved in 0.1% formic acid (FA), and LC‒MS/MS was performed using an UltiMate 3000 Nano liquid chromatography system (Thermo Fisher Scientific, Dreieich, Germany) coupled online to a Q Exactive HF‒X mass spectrometer (Thermo Fisher Scientific, Dreieich, Germany). Separation was performed at 250 nl/min on an EasySpray column (PepMap RSLC C18, 50 cm length, 75 µm ID, # ES903, Thermo Fisher Scientific, Dreieich, Germany). Solvent A was 0.1% FA in water, and solvent B was 0.1% FA in acetonitrile. A 30-minute gradient from 3–25% solvent B followed by a 5-minute gradient from 25–40% solvent B, was used for peptide separation. Spectra were collected via a top-12 acquisition method. For protein identification and quantification, MaxQuant V1.6.1 [23] was used in combination with the human and EBV subsets of the UniProt database. Statistical validation and volcano plot generation were performed using Perseus V1.5.3.2. Enrichment analysis was performed using WebGestalt [10]. The mass spectrometry proteomics data have been deposited into the ProteomeXchange Consortium database via the PRIDE [15] partner repository under the dataset identifier PXD051181.

## Results

### Generation of monoclonal antibodies against monomethylarginine (MMA)-modified EBNA1

Using monoclonal antibodies, we showed previously that Epstein–Barr virus nuclear antigen 2 (EBNA2) contains SDMA, ADMA, and MMA-modified residues within its RGRG repeat, but these antibodies also reacted with the N-terminal RG repeat (LR1) of nuclear antigen 1 (EBNA1) [1]. Therefore, in this study, we generated mAbs against the large C-terminal RGRGG-containing repeat (LR2) of EBNA1 to determine whether these mAbs would recognize EBNA1 as well as similarly modified cellular proteins.

**Fig. 1:**
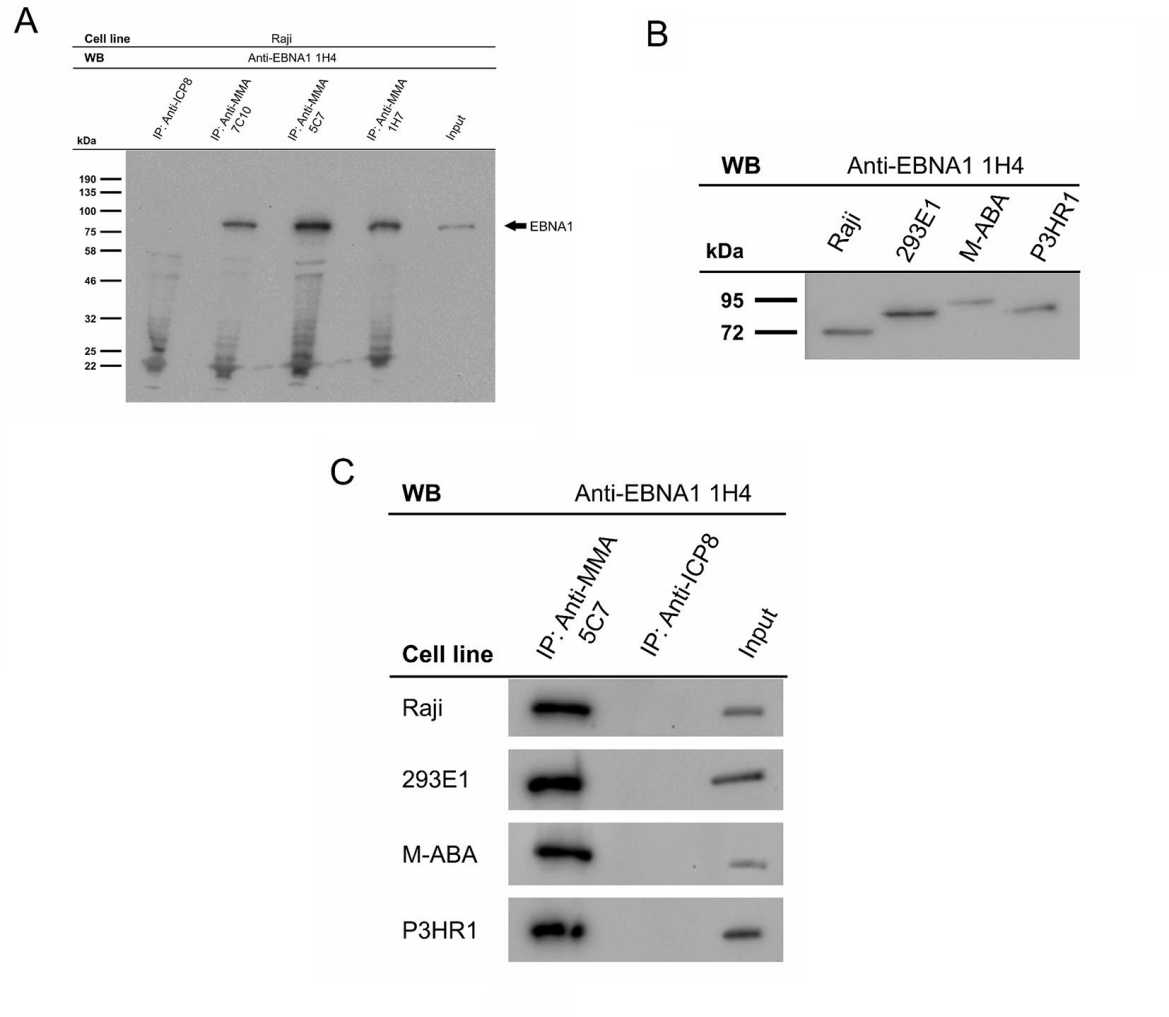
Characterization of mouse monoclonal antibodies (mAbs) directed against MMA-modified EBNA1 via Western blotting. (**A**) Extracts of the BL cell line Raji (EBV-positive) were precipitated with the mAbs 7C10, 5D7, and 1H7 as well as the control antibody “anti-ICP8” (isotype IgG control). Bound EBNA1 was analyzed by SDS-PAGE and visualized by immunoblotting with the rat monoclonal antibody 1H4 and the appropriate horseradish-peroxidase-conjugated mouse anti-rat IgG secondary antibody using enhanced chemiluminescence (ECL). (**B**) Extracts of the indicated cell lines were analyzed by Western blotting. EBNA1 was visualized by ECL after immunoblotting using the rat mAb 1H4 and the appropriate secondary antibodies. The cell lines expressed EBNA1 proteins of different sizes due to strain variability. (**C**) Extracts of the indicated cell lines were used for precipitation with mAb 5C7 or the isotype control antibody “anti-ICP8”. The precipitated EBNA1 was visualized by immunoblotting with mAb 1H4

**Fig. 2:**
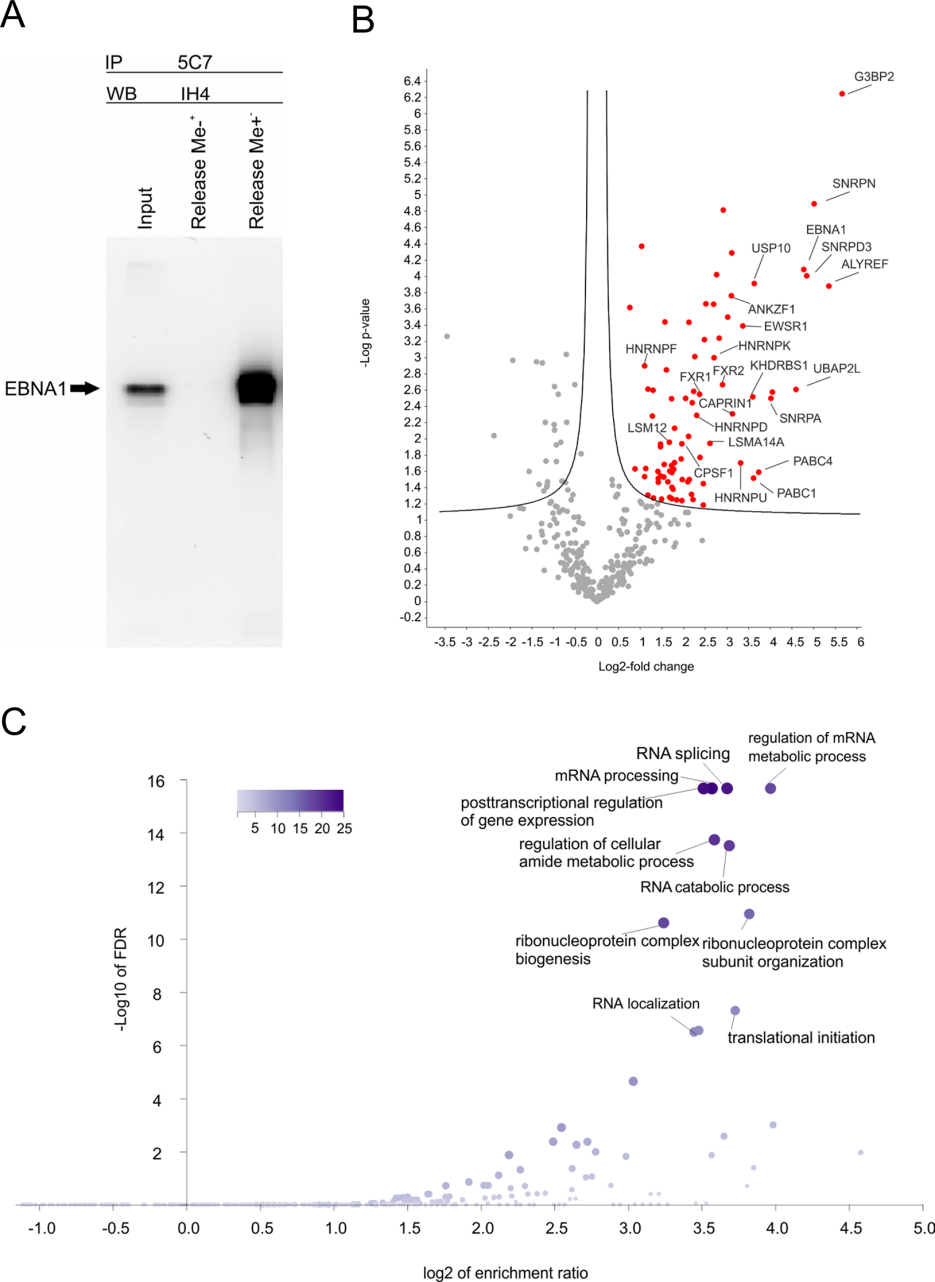
Peptide release of EBNA1 and its interactors. (**A**) Extracts of Raji cells were subjected to immunoprecipitation using the mouse mAb 5C7. The resin was incubated with the peptide RGRGRGG containing either MM-modified arginine residues (“Release Me+”) or unmodified arginine residues (“Release Me-”). The released EBNA1 was visualized by ECL after immunoblotting using the rat mAb 1H4. A 10-µL aliquot of the cell extract was run in an adjacent lane. (**B**) Volcano plot analysis of mass-spectrometry-based label-free quantification (LFQ) values of proteins released with the MM-modified and unmodified RGRGRGG peptides. Proteins that were more abundant in the release experiment with the MM-modified peptide are highlighted in red. (**C**) Overrepresentation analysis of this set of proteins was performed with WebGestalt (www.webgestalt.org). The volcano plot includes the enriched functional categories. The size and color of the dots in the scale indicate the sizes of the gene sets

Clones 5C7, 7C10, and 1H7 were used for immunoprecipitation of EBNA1, as shown in Fig. 1A. Equal loading was confirmed (Supplementary Fig. S2A). mAb 5C7 was used in the subsequent analyses for the precipitation of EBNA1 from EBV-containing Raji, M-ABA, and P3HR1 cells and from B95.8-strain-containing HEK 293E1 cells. The corresponding EBNA1 proteins contain GA repeats of varying length, resulting in slightly different mobility on SDS-PAGE gels, as shown in Fig. 1B. P3HR1 cells harbor type 2 EBV, and the other three lines harbor type 1 EBV. The precipitation of the EBNA1 protein by mAb 5C7 is shown in Fig. 1B, and the corresponding control reactions are shown in Fig. 1C. mAb 5C7 reacted with both peptides used for immunization. We determined that the sequence GRGRGG was the most likely overlapping sequence. We used two peptides, one with, and one without MMA modification, for release experiments. As shown in Fig. 2A, only the MMA-modified peptide was able to release the precipitated EBNA1, indicating that mAb 5C7 recognizes MMA-modified EBNA1. The Raji cell extract was used for immunoprecipitation with mAb 5C7 in a peptide release assay as described above. The proteins from four independent experiments and the corresponding controls (Supplementary Fig. S2B) were analyzed via mass spectrometry. A list of proteins that were identified in the protein release experiments but were not present in the control precipitates is shown in Table 1. EBNA1 was not present in any of the corresponding control precipitates.

**Table 1:** Proteins that were identified in protein release experiments

Gene	Protein name	Oncogenic function (PMID)
SNRPD3	Small nuclear ribonucleoprotein SmD3	
ALYREF	THO complex subunit 4	37537569
EBNA1	EBNA1	
RPS15A	40S ribosomal protein S15a	30661291
DIDO1	Death-inducer obliterator 1	22469980
LSM12	Protein LSM12 homolog	37394011
LSM14A	Protein LSM14 homolog A	
DAP3	28S ribosomal protein S29, mitochondrial	7499268
CPSF1	Cleavage and polyadenylation specificity factor subunit 1	36381317
NACA	Nascent polypeptide- associated complex subunit alpha	
KHDRBS1	KH domain-containing, RNA-binding, signal transduction-associated protein 1	37208334
MIA3	Melanoma inhibitory activity protein 3	37948019
ERH	Enhancer of rudimentary homolog	37997813
PGAM5	Serine/threonine-protein phosphatase PGAM5	33370650
CHERP	Calcium homeostasis endoplasmic reticulum protein	12656674
SLC25A3	Phosphate carrier protein, mitochondrial	
HNRNPCL1	Heterogeneous nuclear ribonucleoprotein C-like 1	34386035
AKAP8	A-kinase anchor protein 8	31980632
RPL29	60S ribosomal protein L29	22868929
SNRPN	Small nuclear ribonucleoprotein- associated protein N	26261020

To identify less prominently enriched proteins, volcano plot analysis was performed (Fig. 2B). The entire set of significantly enriched proteins is listed in Supplementary Table S1. Overrepresentation analysis of this set of proteins revealed significant enrichment of RNA binding or processing proteins (Fig. 2C). RNA binding of EBNA1 via its RG repeats has been demonstrated experimentally [12]. Notably, many of the proteins identified in this study have RG repeats, e.g., SmD3. References (PMIDs) to possible or known roles of the significantly enriched proteins in tumor progression or inhibition or to potential roles in apoptosis are listed in Table 1. The references corresponding to the PMIDs and the known or possible roles of the proteins in tumorigenesis are listed in Supplementary Table S2. For example, DIDO1 promotes the progression of melanoma and inhibits the apoptosis of melanoma cells [3]. In dengue-virus-infected cells, DIDO1 supports virus production by interfering with the interferon response [4]. DIDO1 forms a complex with HNRNPK [17], another confirmed EBNA1 interactor [1] that was also detected in the present study.

## Discussion

Viral proteins often affect cellular regulatory pathways by binding to cellular proteins to interfere with or alter their function. For example, by binding via its TrpTrpPro (WWP) motif, nuclear antigen 2 of EBV (EBNA2) alters the function of the repressor JBPjK to activate otherwise silent genes [11]. Using monoclonal antibodies against SDMA-modified EBNA2 [2], we showed that this modification confers binding ability to the survival motor neuron (SMN) protein, possibly by interfering with SMN’s cellular partner protein DDX20 (DP103/Gemin-3) [24].

As mentioned above, mAbs against MMA-modified EBNA2 reacted not only with EBNA2 and various other cellular proteins but also with EBNA1 [1]. Two of the goals of the experiments presented here were to determine whether EBNA1 contains MMA residues within its C-terminal RG repeat and to identify proteins that share similarly modified surface epitopes. In addition, we predicted that cellular proteins that interact with EBNA1 might be identified by coimmunoprecipitation with mAbs. It is therefore noteworthy that NACA (nascent polypeptide-associated complex subunit alpha), which is one of the proteins shown in Table 1, was shown recently to be an EBNA1-binding protein. The association of EBNA1 with NACA is mediated via the glycine-alanine (GAr) stretch of EBNA1 rather than its RG repeats [29]. Some proteins that were precipitated with antibodies against MMA-modified EBNA2 were also precipitated with the mAbs used in this study: ALY/REF, SNRPD3, and some proteins of ribosomal subunits were identified in both analyses.

Some proteins that reacted only with mAb 5C7 may play a role in EBV-mediated transformation. For example, activation of the proto-oncogene c-MYC via chromosomal translocation is a hallmark of Burkitt’s lymphoma [18]. Three of the proteins identified in this study interact with c-MYC RNA: CAPRIN1 binds c-MYC mRNA via its C-terminal RGG repeats [21]; CPSF1 stabilizes c-MYC mRNA, thus increasing the c-MYC level [13]; and the FXR1 protein, together with FXR2, forms a complex with CAPRIN1 [22]. ALY/REF, in turn, is activated by c-MYC and drives cancer cell proliferation [25].

In summary, we have identified various MMA-modified proteins that bind to the novel antibody 5C7, either directly or indirectly via MMA-modified EBNA1, and these proteins are involved in various cellular functions, including gene regulation, RNA processing, export, transcriptional activation, and splicing. The identification of these interacting proteins will serve as a starting point for future studies of EBV function and EBNA1-associated factors involved in cellular transformation or multiple sclerosis.

## Electronic Supplementary Material

Below is the link to the electronic supplementary material

Supplementary Material 1

Supplementary Material 2

Supplementary Material 3

Supplementary Material 4

## Data Availability

No datasets were generated or analysed during the current study.
