# Supplementary material for: Use of antibodies against Epstein–Barr virus nuclear antigen 1 for detection of cellular proteins with monomethylated arginine residues that are potentially involved in viral transformation
Source: Arch Virol. 2024 Nov 8;169(12):241. doi: 10.1007/s00705-024-06172-7 (PMC11549202; doi:10.1007/s00705-024-06172-7)
Supplement: Supplementary file 4 — Supplementary Material 4 [file 705_2024_6172_MOESM4_ESM.docx]

| **Genes** | **Protein names** | **Oncogenic function** | **References** |
| --- | --- | --- | --- |
| SNRPD3 | Small nuclear ribonucleoprotein SmD3 |  |  |
| ALYREF | THO complex subunit 4 | ALYREF/YAP1 signaling | Yang Q, et. (2023) Mol Cancer 22:122 |
| EBNA1 | EBNA1 |  |  |
| RPS15A | 40S ribosomal protein S15a | Akt/IKK-beta/NF-kB signalling | Liu C, et al. (2019) J Cell Mol Med 23:2207-2218 |
| DIDO1 | Death-inducer obliterator 1 | BMP-induced melanoma | Braig S, Bosserhoff AK (2013) Oncogene 32:837-848 |
| LSM12 | Protein LSM12 homolog | Calcium mobilization | Hessling LD, et al. (2023) Biochim Biophys Acta Mol Cell Res 1870:119531 |
| LSM14A | Protein LSM14 homolog A |  |  |
| DAP3 | 28S ribosomal protein S29, mitochondrial | Interferon-gamma-induced cell death | Kissil JL, et al. (1995) J Biol Chem 270:27932-27936 |
| CPSF1 | Cleavage and polyadenylation  specificity factor subunit 1 | Alternatve polyadenylation (APA) | Kang W, et al. (2022) Am J Cancer Res 12:4566-4583 |
| NACA | Nascent polypeptide-  associated complex subunit alpha |  |  |
| KHDRBS1 | KH domain-containing, RNA-binding,  signal transduction-associated protein 1 | Drug resistance | Wong TL, et al. (2023) Nat Commun 14:2861 |
| MIA3 | Melanoma inhibitory activity protein 3 | **Anti-apoptotic** | Wanbiao Z, et al. (2023) Mol Cell Biochem Epub ahead of print. PMID: 37948019 |
| ERH | Enhancer of rudimentary homolog | EMT | Tsai YM, et al. (2024) Mol Med Rep 29(1):9 |
| PGAM5 | Serine/threonine-protein phosphatase PGAM5 | Mitophagic death signaling | Cheng M, et al. (2021) Eur J Cell Biol 100:151144 |
| CHERP | Calcium homeostasis endoplasmic  reticulum protein | Calacium mediated cell cycle progression | O'Rourke FA, et al. (2003) Biochem J 373:133-143 |
| SLC25A3 | Phosphate carrier protein, mitochondrial |  |  |
| HNRNPCL1 | Heterogeneous nuclear  ribonucleoprotein C-like 1 | Drug resistance | Liu M, et al. (2021) Front Genet 12:669605 |
| AKAP8 | A-kinase anchor protein 8 | EMT-RNA metabolism | Hu X, et al. (2020) Nat Commun 11:486 |
| RPL29 | 60S ribosomal protein L29 | Anti-apoptotic | Li C, et al. (2012) Mol Cell Biochem 370:127-139 |
| SNRPN | Small nuclear ribonucleoprotein-  associated protein N | **Anti-apoptotic** | Ma J, et al. (2015) Mol Med Rep 12:6060-6064 |
